# 

**Published:** 1890-02

**Authors:** 


### BOOKS AND PAMPHLETS RECEIVED.

"*Archives of Veterinary Science,*" St. Petersburg, Russia, Oct. 1889. One hundred and twenty-six pages of official communications and valuble standard articles.

"*Supplement to Archives of Veterinary Science, 1889.*" "A text book on Internal Medicine of Domestic Animals, Relating to the Organs of Motion, Nervous System, Chronic and Constitutional." By F. Friedberg and E. Ferner. Second revised edition, translated into Russian by J. M. Schmulewitch, St. Petersburg.

"Studies from the Biological Laboratory of Johns Hopkins University." Edited by H. Newell Martin, M.D., &c. Vol. IV., No. 5.

"Bulletin de la Societé Zoologique de France." T. XVI., No. 9.

"Annales et Bulletin de la Societé de Médecine de Gand."

"Bulletin de L'Academie Royal de Médecine de Belgique."

"Proceedings of the Canadian Institute, Toronto." Vol. VII., Fas. 1.

"Proceedings Davenport Academy of Natural Sciences." Vol. V., Part 1. 1884–89.

"Il Naturalista Siciliano." Palermo.

"Annalen des K. K. Naturhistorischen Hofrauseums." Band IV., Nos. 2 and 3. Wien, 1889. Edited by Dr. Franz Ritter von Hauer.

"Journal of the Asiatic Society of Bengal." Vol. LVIII., Part II., Nos. 1 and 2. Calcutta, 1889.

